# Assessment of the influence of viscoelasticity of cornea in animal ex vivo model using air‐puff optical coherence tomography and corneal hysteresis

**DOI:** 10.1002/jbio.201800154

**Published:** 2018-10-14

**Authors:** Ewa Maczynska, Karol Karnowski, Krzysztof Szulzycki, Monika Malinowska, Hubert Dolezyczek, Artur Cichanski, Maciej Wojtkowski, Bartlomiej Kaluzny, Ireneusz Grulkowski

**Affiliations:** ^1^ Institute of Physics, Faculty of Physics, Astronomy and Informatics Nicolaus Copernicus University Torun Poland; ^2^ Laboratory of Molecular and Systemic Neuromorphology, Department of Neurophysiology Nencki Institute of Experimental Biology, Polish Academy of Sciences Warsaw Poland; ^3^ Institute of Mechanics and Machine Design, Faculty of Mechanical Engineering UTP University of Science and Technology Bydgoszcz Poland; ^4^ Institute of Physical Chemistry Polish Academy of Sciences Warsaw Poland; ^5^ Department of Optometry, Collegium Medicum Nicolaus Copernicus University Bydgoszcz Poland

**Keywords:** corneal biomechanics, corneal hysteresis, optical coherence tomography, viscoelasticity

## Abstract

Application of the air‐puff swept source optical coherence tomography (SS‐OCT) instrument to determine the influence of viscoelasticity on the relation between overall the air‐puff force and corneal apex displacement of porcine corneas ex vivo is demonstrated. Simultaneous recording of time‐evolution of the tissue displacement and air pulse stimulus allows obtaining valuable information related in part to the mechanical properties of the cornea. A novel approach based on quantitative analysis of the corneal hysteresis of OCT data is presented. The corneal response to the air pulse is assessed for different well‐controlled intraocular pressure (IOP) levels and for the progression of cross‐linking‐induced stiffness of the cornea. Micrometer resolution, fast acquisition and noncontact character of the air‐puff SS‐OCT measurements have potential to improve the in vivo assessment of mechanical properties of the human corneas.

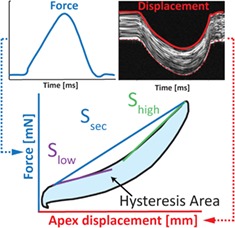

## Graphical abstract

In this study, a novel approach to determine the viscoelasticity of porcine corneas ex vivo based on air‐puff swept source optical coherence tomography is demonstrated. The prototype system allows to measure and analyze the corneal hysteresis quantitatively. The viscoelastic response of the cornea to air puff is determined for different intraocular pressure levels and different tissue stiffness values.

## 1 INTRODUCTION

Mechanical functions and viscoelastic properties of the cornea depend mainly on the unique composition and architecture of the collagen fibrils constituting that tissue 1, 2. Information about the structure and biomechanics of the cornea is the foundation of diagnosis and management of ocular diseases such as keratoconus. It can also help in the proper planning of surgical procedures, detecting contraindications and in the monitoring of the healing process 3. Expanding knowledge about corneal biomechanics is also extremely important for the advances in the reliable evaluation of the intraocular pressure (IOP), thus for more effective diagnosis and treatment of glaucoma 4.

The reaction to mechanical stimulus forms a basis for the studies of biomechanical properties of the corneal tissue. However, corneal dynamics due to the applied load is a complex effect, resulting from the force balance between to the biomechanics and the IOP. Therefore, biomechanical fundamentals of tonometry (IOP measurement), as well as corneal geometry (thickness and curvature), have a significant impact on the accuracy of the IOP determination. The corneal behavior is governed by its viscoelastic properties. It means that when the force is removed, part of the energy delivered to such a viscoelastic tissue during loading is dissipated, and the return is delayed in time, which manifests as a hysteresis 2, 5.

The need for understanding the mechanical properties of the tissue in its natural conditions paved the way for the development of a new medical imaging modality—elastography 6, 7, 8, 9, 10. Generally, in elastography, information on biomechanical properties is extracted from the displacement of the tissue due to its mechanical loading 8, 9. Combination of mechanical excitation and visualization of tissue reaction to the load was pioneered with the imaging technologies like ultrasound 6 and magnetic resonance 11. Later on, optical modalities such as Brillouin microscopy 12, Scheimpflug imaging 13, 14 or atomic force microscopy 15, enabled mapping the biomechanical properties of the tissue at different organizational levels and scales.

Optical coherence tomography (OCT) is a noninvasive, high‐speed, high‐resolution optical imaging technology based on low‐coherence interferometry. Nowadays, OCT enables three‐dimensional (3‐D) reconstruction of the structure of the biological object with micrometer resolution for early ocular diseases diagnosis and treatment monitoring 16, 17, 18. In the newest generation of OCT, called swept source OCT (SS‐OCT), the wavelength tunable laser is utilized, and the interferometric signal is acquired in time.

Elastography based on OCT, referred to as optical coherence elastography (OCE), is particularly attractive since it enables imaging at the micrometer resolution and has the potential for early detection of the pathology. OCE dedicated to the evaluation of the corneal elastic properties includes different mechanical excitation mechanisms. The load may be applied in a contact or noncontact way, for example, by the gonioscopy lens 19, mechanical indentation 20, 21, pulsed laser 22, sound waves 23, airborne ultrasound 24 and the air puff from the tube or needle 25, 26, 27. The performance of the OCT‐based elastography has been demonstrated in in vitro corneal tissues 28, in ex vivo eye models 19, 29 as well as in in vivo studies with patients 25, 26. OCT allowed to monitor the geometrical changes of the cornea due to the increased IOP. Speckle analysis was also utilized during deformation to extract information on micrometer displacement. What is more, phase sensitive OCT was applied to perform elastic wave velocity imaging 30, 31, 32.

The air puff constitutes the only excitation scenario that is currently accepted clinically and widely used in noncontact tonometry devices. The studies demonstrating the integration of corneal stimulation by air puff with OCT imaging utilized SS‐OCT and spectral‐domain OCT technology and enabled visualization of the apex deformation in time (M‐scan) or the dynamics of the selected cross‐section of the cornea (repeated B‐scan) 25, 26. The measurements were performed for in vivo human eyes and ex vivo porcine eyes with the analysis of the deformation, thickness or displaced volume. Later on, Karnowski et al combined the corneal displacement directly measured with OCT with the temporal profile of the applied air pressure to generate hysteresis loop 33.

The aim of this study is to apply swept source optical coherence tomography combined with the air‐puff system (air‐puff SS‐OCT) to assess rapid dynamics of porcine corneas during the air pulse application. We demonstrate a novel approach based on a generation of dynamic corneal hysteresis (CH) proving that it can be a direct signature of the tissue viscoelasticity. ex vivo measurements with porcine eye model enable full control of the IOP and provide the opportunity to alter also mechanical properties (stiffness) of corneas under investigation (eg, via collagen cross‐linking [CXL] procedure). The study design enables comprehensive examination of the relation between IOP and corneal stiffness and the impact of those factors on the parameters of the hysteresis curve.

## 2 MATERIALS AND METHODS

### 2.1 The experimental set‐up

**Figure 1:**
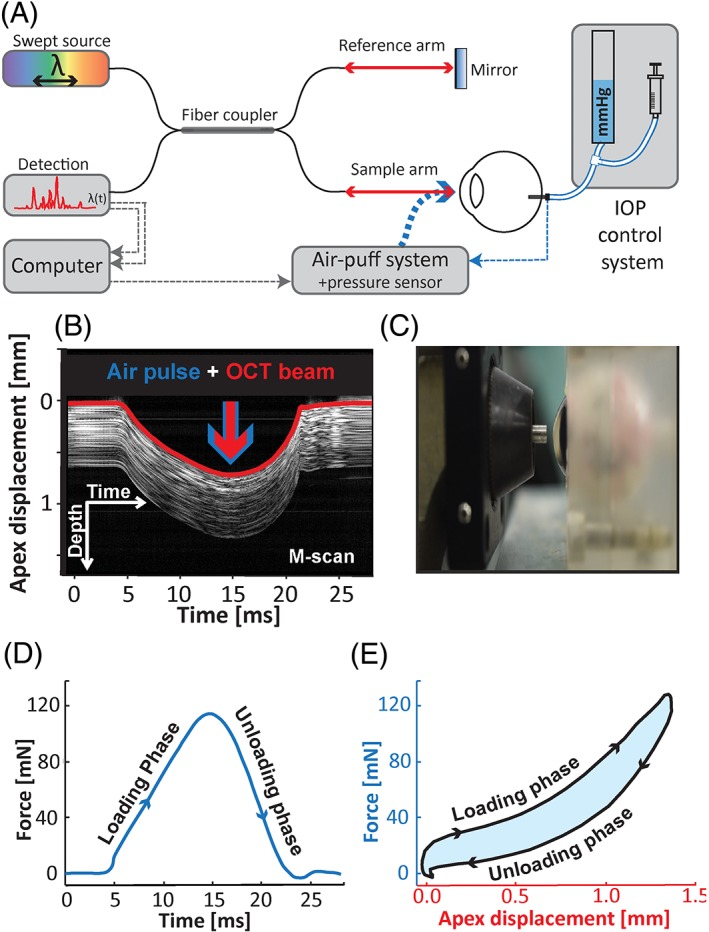
Simplified schematic diagram of the air‐puff SS‐OCT instrument. (A) Swept‐source OCT set‐up combined with air puff and IOP control systems. (B) SS‐OCT M‐scan (composed of 1600 A‐scans) of the cornea during air‐puff stimulation. Temporal deformation of anterior corneal surface in marked with red curve. (C) A photography of the porcine eye placed in front of the air‐puff chamber. (D) Temporal profile of the force applied on the cornea during measurement. (E) Dynamic hysteresis curve for a single air‐puff stimulation

In this study, we evaluated the corneal biomechanics of ex vivo porcine eyes at well‐controlled IOP conditions. The system consisted of three modules: a custom SS‐OCT set‐up, an air‐puff system and an IOP‐control system (Figure 1A). The SS‐OCT system used a swept light source with a central wavelength of 1310 nm and a tuning range of 100 nm at 50 kHz wavelength sweeping rate (Axsun Technologies Inc., Billerica, Massachusetts). The axial and transverse resolutions were 19 and 30 μm in the air, respectively. The available depth range of the air‐puff SS‐OCT instrument was 9 mm in the air.

The air‐puff chamber from commercial tonometer (XPert NCT; Reichert Inc., Depew, New York) was adopted and integrated into sample arm of the OCT setup. The optical axis of SS‐OCT and the direction of the air stream were set to be collinear 25. During each measurement, the M‐scan consisting of 1600 A‐scans from the central point of the applanation area was acquired along with the temporal profile of the air pressure generated by the air‐puff chamber (Figure 1B,D). The latter one was measured with an incorporated pressure sensor (SenSym, Milpitas, California). The pressure at the peak of the temporal profile was measured to be 18.18 ± 0.48 kPa, which corresponded to 120.8 ± 3.2 mN (measured with a custom force measurement system). To calibrate custom force sensor, we used a strain gauge (sensing area of ~700 mm^2^) that was precalibrated in the horizontal position with a set of well‐defined weights (1‐10 g). Next, the readouts from the internal pressure sensor and the gauge placed vertically in front of the exit tube of the air‐puff chamber were recorded while the voltage at the capacitor actuating the air‐puff chamber solenoid was varied. The force exerted by the air puff, which was measured simultaneously with a strain gauge and an internal pressure sensor, enabled finding relationships between the signal from the internal pressure sensor and the air‐puff force. Finally, the temporal profiles of the corneal deformation and air‐puff force were used to generate the CH curves (Figure 1E).

We stabilized eyeballs using a custom holder (Figure 1C). Moreover, the sample was placed in an acrylic glass chamber to provide controlled environmental conditions during the measurements (ie, humidity 65% ± 5% and temperature 21°C ± 2°C). The distance between the cornea and the exit pipe of the air‐delivery system was set to be 3 mm. The pressure inside the eye was exerted by means of the height of the saline column, which was controlled manually with the syringe (Figure 1A) and monitored by the dedicated pressure sensor (Sen‐02, Experimetria, Hungary). The needle for the IOP control was inserted through the sclera into the vitreous body in the back of the eye.

### 2.2 Preparation of the porcine eyes

Sixty‐one freshly enucleated porcine eyes were used in this study. The samples were obtained from a local slaughterhouse and measured postmortem within 8 hours. The eyes immersed in saline solution were kept in a refrigerator before the measurement. Only the eyes with intact cornea and no sign of edema were selected for the experiment.

### 2.3 Cyclic changes of IOP levels

First, we investigated the corneal response to the air pulse under different IOP levels. The total number of 35 porcine eyes were examined in the cyclic inflation test. The cycle with 5 mm Hg step and 30 mm Hg span consisted of two phases: ascending IOP phase (IOP increasing from 5 to 35 mm Hg) and descending IOP phase (IOP decreasing from 35 to 5 mm Hg). The cycle span was chosen to cover the entire range of physiological IOP.

### 2.4 Alteration of corneal stiffness

Next, the tissue response on the air pulse was measured under different corneal stiffness conditions. We altered corneal biomechanics while keeping the IOP constant by performing CXL of corneal collagen—a procedure used in standard clinical practice to strengthen keratoconic corneas by modification of collagen architecture 34, 35, 36. We performed CXL according to the Dresden protocol that was adjusted for porcine corneas, which are thicker than human ones 37. Once the epithelium was removed (from the central 8 mm diameter area), the cornea was treated with the riboflavin solution (RS) (0.1% riboflavin and 20% dextran; Ricrolin, Sooft Italia; 2 drops/3 min) for 30 minutes to improve the efficiency of photosensitizer penetration through the cornea. For the next 30 minutes, the RS was applied as previously (2 drops/5 min) and the sample was simultaneously illuminated with a UV‐A lamp (XLink‐Corneal Crosslinking System; Opto Electronica S/A, Brazil; 365 nm; 3 mW/cm^2^ corresponding to 5.37 J/cm^2^).

This part of the study was performed on 13 pairs of porcine eye models (26 eyes in total). One eye of each pair underwent CXL procedure, whereas the fellow eye had only epithelium removed and served as a control. The corneal response to the air puff was determined at different stages: after epithelium removal (EPI OFF), after saturation with RS and immediately after complete CXL treatment (ultraviolet [UV] + RS). In each case, five consecutive measurements were performed under physiological and elevated IOP levels of 15 and 25 mm Hg, respectively.

### 2.5 Analysis of CH

**Figure 2:**
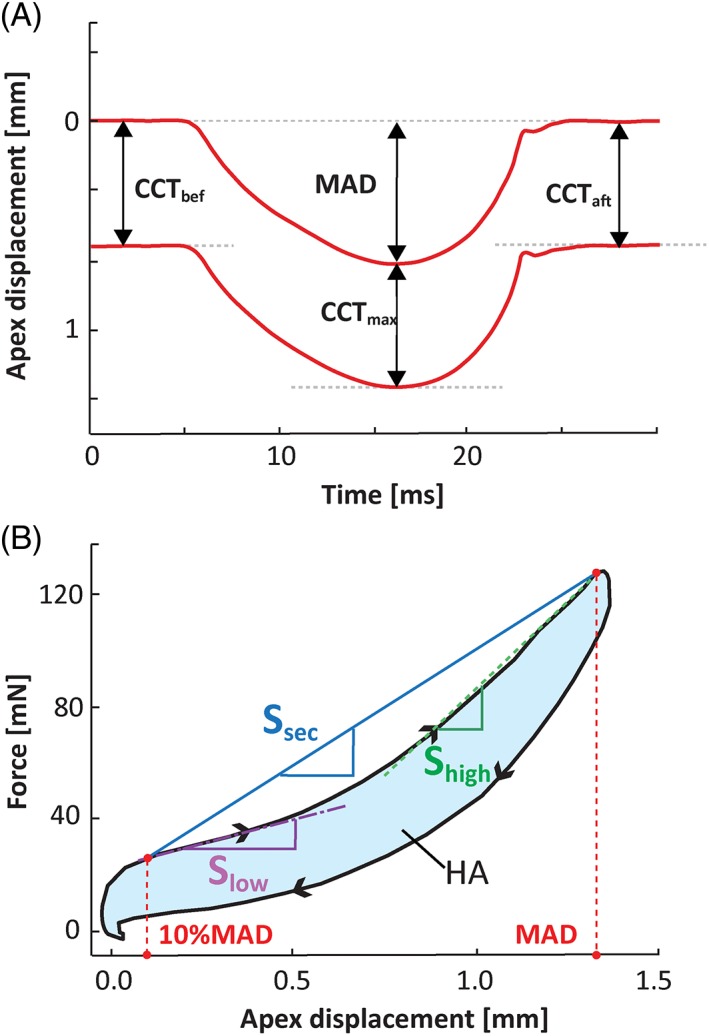
Parameters extracted from the measurements: (A) After segmentation of the corneal surfaces, the information about the maximum displacement of the apex (MAD) as well as the central corneal thickness before (CCT_bef_), at maximum displacement (CCT_max_) and after the puff (CCT_aft_) application were extracted; (B) Secant slope (*S*_sec_), high‐strain slope (*S*_high_), and low‐strain slope (*S*_low_) were determined based on the loading curve of hysteresis. The area enclosed by the HA is also calculated. Graphical description of *S*_low_ and *S*_high_ is conceptual (see detailed description in Section 2.6)

In order to reconstruct the hysteresis curve, the anterior surface of the corneal SS‐OCT M‐scans was segmented to plot the apex displacement varying in time (Figure 1B). Pressure wave readings from the pressure sensor were converted into force wave (Figure 1C). Temporal traces of corneal apex displacement *x*(*t*) and force wave *F*(*t*) were combined as a hysteresis curve *F*(*x*) for each dataset (Figure 1D and 2B). To analyze central corneal thickness (CCT), the posterior surface of the corneal SS‐OCT M‐scans was also segmented (Figure 2A). CCT was measured at three instances during corneal deformation and recovery process, that is, before air pressure applied (CCT_bef_), at the time of maximum displacement (CCT_max_) and after corneal recovery (CCT_aft_). The maximum apex displacement (MAD) was extracted from the anterior surface of the cornea (Figure 2A).

Loading (deformation) and unloading (recovery) phases of the corneal response can be identified in the hysteresis plot (Figure 2B). A part of the energy delivered via the air pulse is dissipated through viscous losses, whereas the remainder is used to recover cornea to its original state. We calculate the hysteresis area (HA) with the following formula:

(1) $$\mathrm{HA}=\int_{0}^{\mathrm{MAD}}F_{\text{loading}}\mathrm{dx}-\int_{\mathrm{MAD}}^{0}F_{\text{unloading}}\mathrm{dx},$$

where *F*_loading_(*x*) and *F*_unloading_(*x*) are abovementioned phases of the hysteresis (Figure 2B). The HA represents the energy loss due to the effect of the viscous properties of the cornea with possible additional contribution from the other structures of the anterior segment and the IOP. The fraction of the mechanical energy loss during a single deformation‐recovery cycle, which we termed here a hysteresis ratio (HR), is calculated by the following equation:

(2) $$\mathrm{HR}=\frac{\mathrm{HA}}{\int_{0}^{\mathrm{MAD}}F_{\text{loading}}\mathrm{dx}}\times 100\%.$$

Based on bilinear nature of the loading curve, we introduced the following parameters describing the corneal deformation process, influenced, among other factors, by the elastic properties of the cornea: high‐strain slope (*S*_high_), and low‐strain slope (*S*_low_) for two linear sections of the curve, and secant slope (*S*_sec_). The *S*_sec_ was calculated as a slope of a line between 10% of MAD point and the MAD point. The *S*_low_ was defined as the slope of the linear fit of the first 120 μm from 10% of MAD point of the loading curve (at low‐strain levels), whereas the *S*_high_ was the slope of the linear fit to the last 120 μm in the loading curve (at high‐strain levels) (Figure 2B).

The results were presented as a mean ± SD. The two‐sample Student's *t* test was used to detect the statistical differences between the OCT measurements. We considered *P*‐values lower than significance level *α* = 0.05 to reject null hypothesis and to obtain statistical significant difference between the means. The correlations between analyzed parameters were assessed using Pearson's coefficient (R).

## 3 RESULTS

### 3.1 Cyclic changes of IOP levels

**Figure 3:**
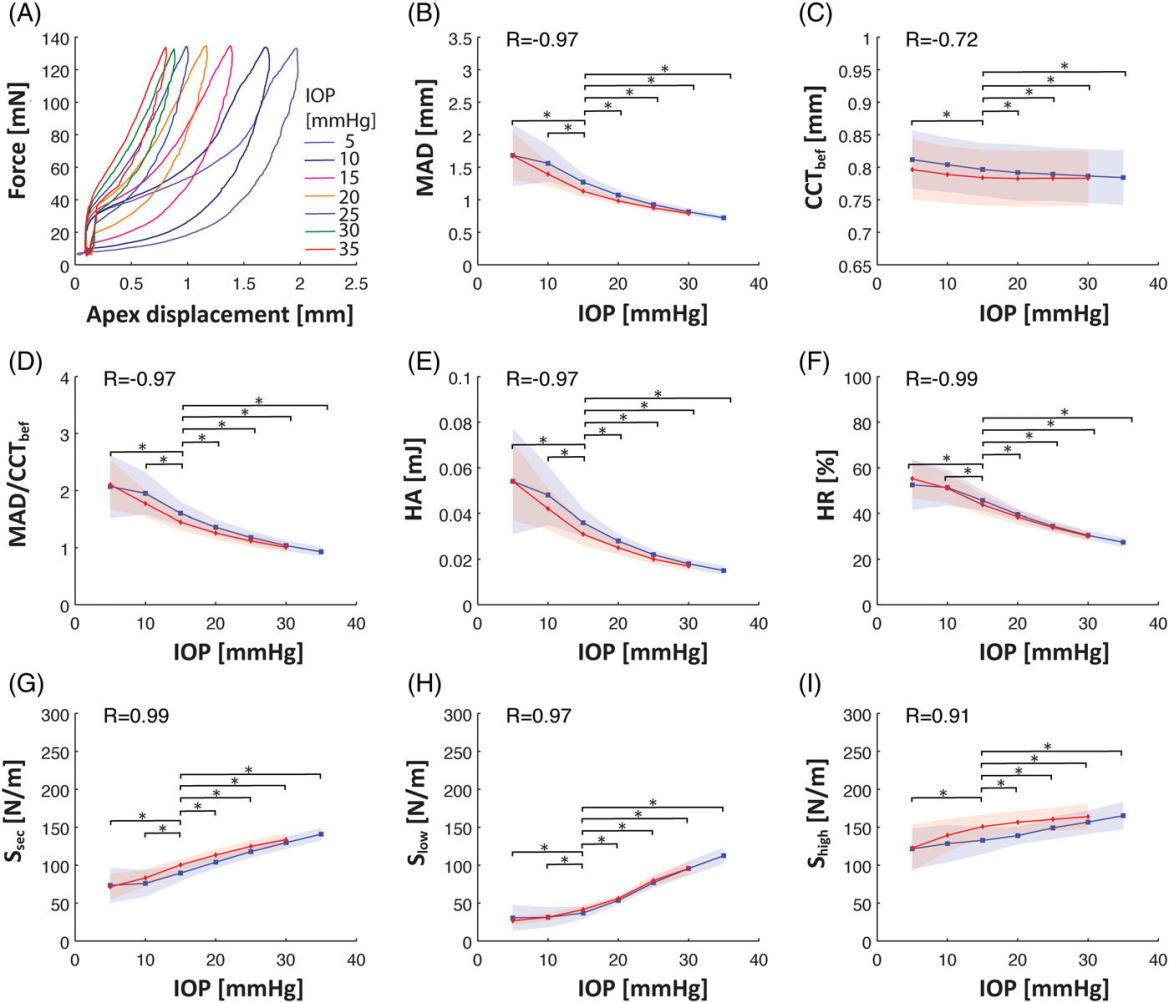
Impact of the IOP on the extracted parameters of hysteresis curve. Results for measurements on 35 porcine corneas ex vivo. (A) Evolution of the hysteresis shape under different IOP levels (for simplicity hysteresis curves only for ascending IOP phase of the cyclic inflation test are presented), (B) MAD, (C) central corneal thickness measured before the air puff (CCT_bef_), (D) CCT‐corrected MAD (MAD/CCT_bef_), (E) HA, (F) HR, (G) secant slope (*S*_sec_), (H) low‐strain slope (*S*_low_), (I) high‐strain slope (*S*_high_) for ascending and descending IOP. Analysis results for ascending phase of hysteresis plot is presented with blue line and points, while descending phase is marked with red color. Errors are presented as shaded plots with width of ±SD. Asterisks indicate statistically significant differences between two analyzed groups. *R* is a Pearson's correlation coefficient

The results in Figure 3 revealed a strong correlation between extracted biomechanical properties of the corneas and IOP levels (Pearson coefficient *R* ~1). Significant changes in dynamic CH curves were observed (Figure 3A). As expected, MAD values decreased with increased eye pressure (Figure 3B). The differences in deformation magnitudes, for IOP levels compared to the physiological level of 15 mm Hg, were statistically significant. CCT measured before the air‐puff stimulus was significantly smaller for higher IOP values (Figure 3C). However, no statistically significant differences in CCT before and after deformation‐recovery were detected. When the deformation force is no longer present, the cornea returns to its original condition, indicating that the air‐puff stimulus does not lead to permanent tissue compression. There was a slight compression of the corneal tissue at the instance of maximum deformation.

The specific J‐shape of the hysteresis curve varied when IOP changed (Figure 3A). Therefore, extracted hysteresis parameters were also affected by different pressure levels (Figure 3E‐I). The HA decreased with increasing IOP (Figure 3E) and was highly correlated with MAD (*R* = 0.9998). A similar trend was observed for HR (Figure 3F). All slopes calculated from the hysteresis increased with the IOP (Figure 3G‐I). The rise of *S*_low_ was more pronounced for IOP levels >15 mm Hg (Figure 3H). The range of measured values for the tested IOP range was the lowest for *S*_high_ (~1.14 N/[m·mm Hg]) among all extracted slopes (Figure 3I). *S*_sec_ was increasing uniformly over entire IOP range (Figure 3G). We found the differences of the secant slope values with respect to IOP = 15 mm Hg to be statistically significant.

Nearly all extracted parameters, except HR and *S*_low_, had different values for the same IOP levels, but in different phases of cyclic inflation test. The high‐strain slope *S*_high_, for instance, had higher values for descending phase of the test cycle. This kind of hysteresis is due to eye inflation in contrary to dynamic hysteresis due to air‐puff stimulus. The “inflation” hysteresis test is performed only on ex vivo eye samples. We, however, focused on the “dynamic” hysteresis as a method that can be applied in vivo.

### 3.2 Alteration of corneal stiffness

In order to investigate the impact of corneal biomechanical properties on the extracted parameters of the hysteresis curve, we performed the measurements for different stiffness conditions. We measured hysteresis curve parameters at different stages of the CXL treatment, that is, after epithelium removal (EPI OFF), after RS application and after UV treatment (RS + UV). The stage after epithelium removal is treated here as a baseline under the assumption that epithelium layer has limited, if any, influence on the corneal stiffness 38.

**Figure 4:**
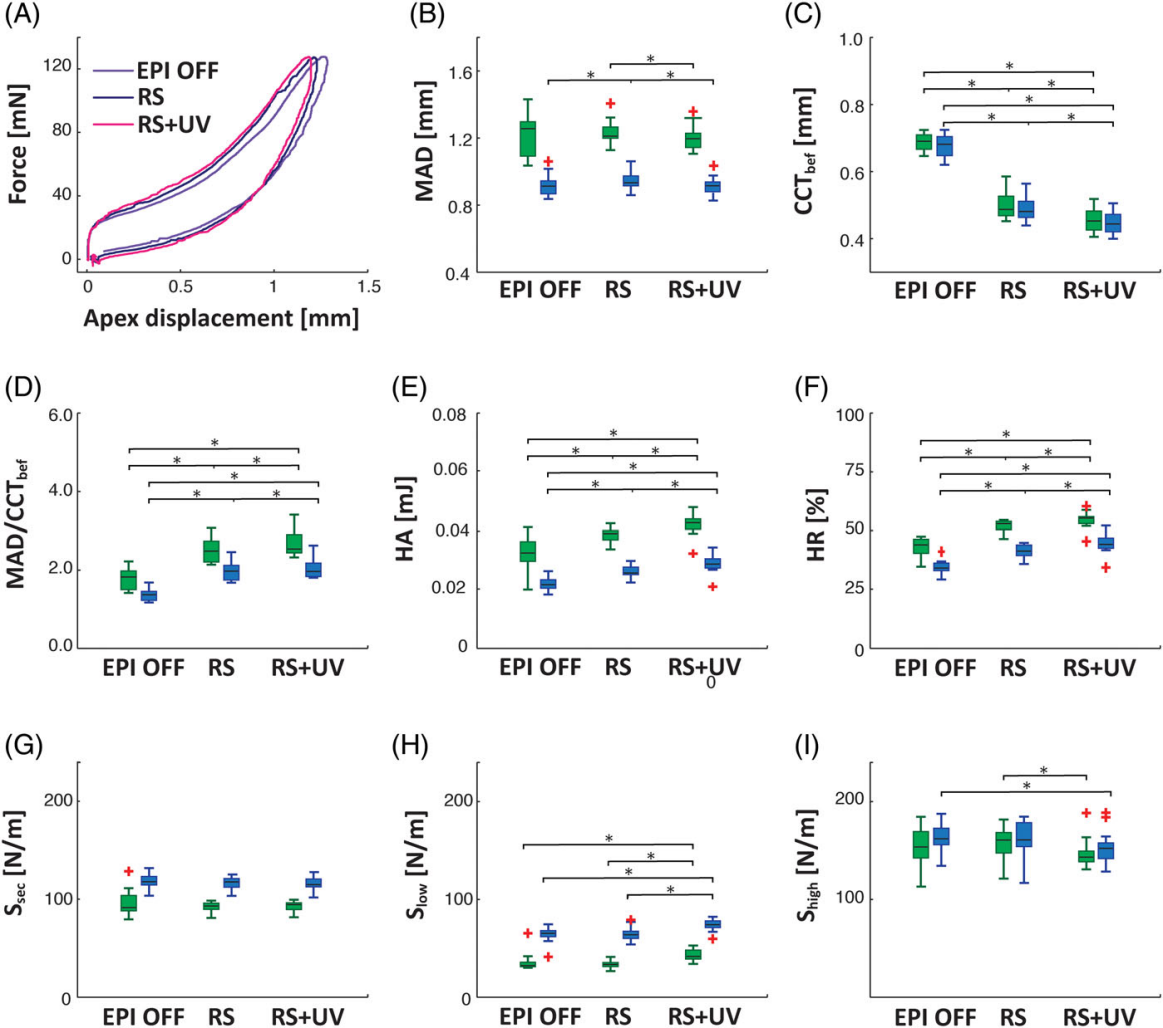
Impact of CXL treatment on extracted parameters of hysteresis curve. Results for measurements on 13 porcine corneas ex vivo. (A) Hysteresis curves at normal IOP of 15 mm Hg for different stages of collagen cross‐linking procedure. (B) MAD, (C) central corneal thickness measured before air puff (CCT_bef_), (D) CCT‐corrected MAD (MAD/CCT_bef_), (E) HA, (F) HR, (G) secant modulus (*S*_sec_), (H) low‐strain slope (*S*_low_), (I) high‐strain slope (*S*_high_) measured at different stages of CXL procedure: after epithelium removal (EPI OFF), after RS saturation and after UVA light irradiation with RS application (RS + UV) under for two IOP levels. Results for IOP = 15 mm Hg were presented with green boxplots, while for 25 mm Hg with blue ones. Red crosses represent outliers

The MAD at every stage of CXL procedure was lower for higher IOP, which correlates with results from the previous section. Statistically significant differences in MAD for RS and RS + UV stages were observed for both pressure levels (Figure 4B). Interestingly, the differences in maximum deformation amplitude after EPI OFF and after RS were statistically significant only for elevated IOP. As expected the CCT decreased by approximately 36% through the whole CXL procedure (Figure 4C). Moreover, statistically significant differences between CCT measured at each stage of CXL procedure at both IOP levels. To account for the changes in corneal thickness, we introduced a new parameter—a ratio of MAD to CCT. Such CCT‐corrected MAD was increasing with the progress of CXL procedure (Figure 4D). The statistical test revealed differences in this new parameter for all stages of CXL and at both IOP levels.

The changes in J‐shaped hysteresis are less prominent than during IOP cyclic test (Figure 4A). HA and HR became statistically larger (Figure 4E,F). Interestingly, the secant slope *S*_sec_ did not change throughout CXL treatment, while some changes were observed for *S*_low_ and *S*_high_ revealing increase and decrease, respectively (Figure 4G‐I). We found statistically significant differences between *S*_high_ measured for RS and RS + UV stages at 15 mm Hg as well as between EPI OFF and RS + UV stages at 25 mm Hg.

## 4 DISCUSSION

In this study, we demonstrated the assessment of biomechanics of the corneas of the porcine eye model ex‐vivo using SS‐OCT system combined with the air puff. The system was able to visualize directly a specific time‐dependent behavior of the cornea (viscoelastic material) under the stress in the form of the hysteresis curve. Most commonly used system, utilizing air puffs (ORA; Reichert Technologies), measures the corneal deformation indirectly be means of corneal reflex of an infrared light 39. Most recent clinical system (Corvis ST, Oculus Optikgeräte GmbH, Germany) uses Scheimpflug technology and measures the corneal deformation directly in a similar way as our air‐puff SS‐OCT. 40, 41 We showed, however, that our system provides superior image quality comparing to Scheimpflug camera‐based Corvis device 42. The parameter called CH measured with ORA is defined as a difference in air‐puff pressures accompanying inward and outward applanation events during stimulation, and it should not be identified as the hysteresis analyzed in this study 39. Wang et al used high‐speed Scheimpflug imaging along with pre‐measured force wave to determine the hysteresis in keratoconus patients 41. However, the proposed methodology did not take into account the movement of the whole eye globe that was reported for such in vivo measurements 43.

Generation and quantitative description of the hysteresis curves enabled extraction of parameters that are related to both elastic and viscous properties of corneal tissue. In particular, corneal elasticity could be characterized by the determination of stress‐strain relation. General description of the corneal reaction with respect to applied force was provided by secant slope *S*_sec_. The loading curve obtained during the increase in the stimulation force demonstrated a typical J‐shape with two characteristics linear regions. The region at lower deformation levels is related to mechanical response dominated by the extracellular matrix (ECM) while collagen fibrils are uncrimping and reorienting under the applied load. The linear phase for higher deformations can be associated with the stretching of the collagen 2, 44, 45. *S*_high_ related to the collagen phase reached higher values than *S*_low_, which indicates that the cornea behaved as a stiffer material in the second phase.

Viscous properties of the cornea could be related to the HA, which indicated the energy dissipated during air‐puff stimulation and was expressed in absolute units (of the order of tens of mJ). Since HA depended on the maximum force, we also included the parameter called HR that described the fraction of the stored energy (during loading) that was dissipated through viscous losses. Generally, less than 50% of stored energy was dissipated during unloading phase. It is important to point out that the force exerted by the air puff and measured by the sensor was not evenly distributed along the corneal surface. However, the puff was mostly concentrated at the center so that the practically the entire force acted on the corneal apex which was the point of deformation measurement. Thus, the HA was a good indicator of dissipated energy.

The results confirmed that the IOP influenced the corneal response to the dynamic external force. Strong correlations between most of the proposed parameters and the IOP were obtained. The MAD depended strongly on the applied IOP (MAD was lower for higher IOP), which follows previous reports 10, 25. It is important to point out that the secant slope *S*_sec_ increased proportionally with the IOP. That effect was observed also in other studies 45, 46, 47, 48. Additionally, the range of values of *S*_low_ obtained between 5 and 35 mm Hg were approximately twice higher than the range of values of *S*_high_ (~44 vs ~82 N/m, respectively), which suggests that the collagen fibers are more rigid than ECM. Viscosity factor represented by the HA was inversely proportional to the material stiffening caused by the IOP changes during inflation test.

CXL enabled microstructural changes of the corneas. The collagen architecture modified by CXL affects both viscous and elastic properties of the cornea although decoupling the impact of those both trends can be hard 14, 49. Proper interpretation of the results of CXL experiment can be done when corneal thinning is taken into account. The changes in CCT were observed after RS saturation and after UV irradiation. The CCT was thinner by ~34% once the entire CXL treatment was completed. A similar effect was observed in both in vivo as well as in vitro studies 13, 29, 50. It is most likely that this effect was associated with the corneal dehydration caused by dextran 51. The limitation of the study is that the measurements with dextran‐only controls have not been included. Moreover, the results of the CXL experiments performed for IOP = 15 mm Hg and IOP = 25 mm Hg were consistent with the data obtained from the inflation test. The MAD became higher after RS saturation and then slightly decreased in comparison with the sample before the treatment. Those results correlated with the observation made by Dorronsoro et al and Kling et al. 10, 26 However, the reported effect of CXL was more pronounced than that in our study. The only parameter that did not show any statistically significant changes during CXL treatment was the slope *S*_sec_. However, corneal thinning effect during the procedure is the confounding factor in the analysis of corneal behavior. Corneal stiffening seems to be masked by the reduction of corneal thickness. Hence, corneal thinning generates limitation of the study. Although we used a standard riboflavin‐UVA induced CXL, the treatments with other agents not causing corneal thinning could be used 52, 53. Since *S*_sec_ showed dependence on the IOP, *S*_sec_ can be also regarded as the potential indicator of the clinically relevant eye parameter like IOP, and therefore can be considered during development of novel noncontact tonometry method. Further in vivo studies with human subjects need to be performed to confirm accuracy and reliability of this IOP estimation.

It is possible to gain more insight into the results of our work by considering a simple viscoelastic model (eg, Kelvin‐Voigt) of the cornea 54. The details of simulations are included in Supporting Information Figure S1. The results of simulations with increasing coefficient of elasticity *k* shown in Figure S1A demonstrated a qualitative correspondence with inflation test results (Figure 3), which indicated that the cornea along with the entire eyeball became more and more rigid as the IOP was increased. The same force level generates lower deformations for higher IOP values. What is more, the simulations presented in Figure S1B corresponded qualitatively to the results of the CXL experiment (Figure 4). Although we expected more significant impact of CXL treatment on the extracted parameters, the results may suggest that the viscous response dominated the observed effects. In fact, it is possible that corneal dehydration during CXL procedure (lower water content of the tissue) impacted its biomechanical properties during the progress of CXL treatment 10, 55, 56.

## 5 CONCLUSIONS

In conclusion, we demonstrated the application of the air‐puff SS‐OCT instrument to determine the influence of the viscoelasticity of ex vivo porcine corneas on the hysteresis curve of overall the air‐puff force as a function of corneal apex displacement. The hysteresis curve properties depended on the pressure inside the eye as well as stiffness of the tissue. The IOP variations caused highly correlated changes in the descriptors of the hysteresis curve, and the porcine eyeballs behave as more rigid bodies for increased IOP. On the other hand, biomechanical modifications of the corneal stiffness due to the CXL seemed to be dominated by the viscosity factor related to strong dehydration of tissue. The secant slope *S*_sec_ can potentially serve as a good parameter to determine the IOP. Micrometer resolution, fast acquisition and noncontact character may make air‐puff SS‐OCT highly attractive for investigation of the human corneas.

## AUTHOR BIOGRAPHIES

Please see Supporting Information online.

## Supporting information


**Author Biographies**

**FIGURE S1** Numerical results of corneal movement using Kelvin‐Voigt model of corneal viscoelasticity for different coefficients of elasticity *k* (A), and for different damping coefficients *c* (B).
