# Supplementary material for: Assessment of the influence of viscoelasticity of cornea in animal ex vivo model using air‐puff optical coherence tomography and corneal hysteresis
Source: J Biophotonics. 2018 Oct 14;12(2):e201800154. doi: 10.1002/jbio.201800154 (PMC7065616; doi:10.1002/jbio.201800154)
Supplement: Supplementary file 1 — Author Biographies [file JBIO-12-e201800154-s001.docx]

| 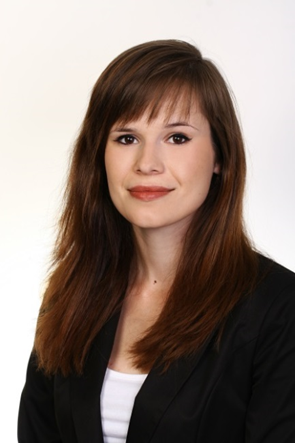 | **Ewa Mączyńska** (born 1989) received her MSc in medical biotechnology from Nicolaus Copernicus University (NCU), Torun, Poland. She is currently a PhD student at the Institute of Physics, NCU. She specializes in biomedical imaging, ophthalmology and signal / image analysis. Her research interests include applications of optical coherence tomography in the assessment of corneal biomechanics and in the precise metrology of contact lenses. |
| --- | --- |
| 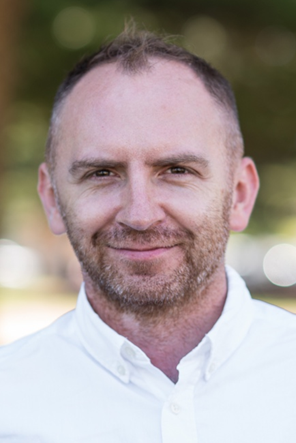 | **Karol Karnowski** received his MSc (Computer Metrology - 2005) and PhD (Biophysics – 2013) from the Nicolaus Copernicus University, Torun, Poland. Since 2015 he is a Research Fellow at Optical + Biomedical Engineering Laboratory, University of Western Australia. The scientific interests of Dr. Karnowski focus on biomedical imaging techniques e.g. optical coherence tomography (OCT) methods and its extensions (OCT angiography, polarization sensitive OCT or OCT angiography). Recently he has been gaining expertise in manufacturing of fiber-based imaging probes from low-resolution upper airway endoscopes to ultrahigh resolution probes used in OCT and Raman spectroscopy. Dr. Karnowski published 14 papers in peer-reviewed journals and is an author of a book chapter. |
| 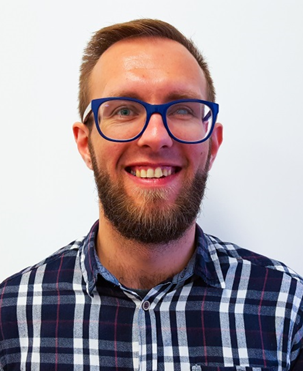 | **Krzysztof Szulzycki** is a PhD student of Biophysics at the Nicolaus Copernicus University in Torun, Poland. He is specialized in acousto-optics and imaging systems like Confocal Microscopy, Optical Coherence Tomography and Optical Coherence Microscopy. He specifically works to develop fast axial scanning with the acousto-optic lens and to apply non-diffractive beams or vector beams in microscopic imaging. |
| 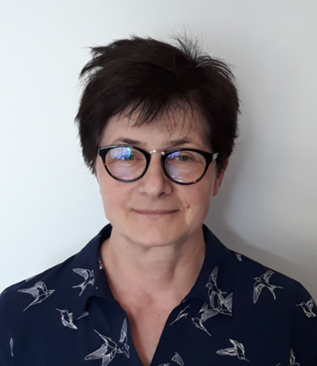 | **Monika Malinowska** works at the Nencki Institute of Experimental Biology in Warsaw, Poland, where she obtained a PhD degree in the field of neuroanatomy. Currently, she is involved in the assessment of structural changes in brain neuroglia in mice developing heart failure as well as in the application of Optical Coherence Microscopy imaging to study the mouse brains in various ischemia models (photostroke or global ischemia). |
| 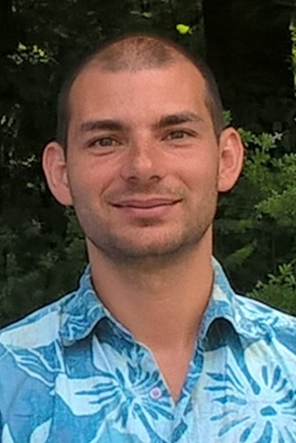 | **Hubert Dolezyczek** is a researcher at Nencki Institute of Experimental Biology, Polish Academy of Sciences in Warsaw, Poland. He received BSc degree from Warsaw University of Life Sciences (2006) and MSc degree in biology from the Jan Kochanowski University in Kielce (2009), Poland. He started his research work at Mossakowski Medical Research Centre Warsaw, Poland where he obtained his microsurgical skills. His research interests focus on developing the methods of rodent brain imaging in vivo with Optical Coherence Microscopy, and histological evaluation of brain under physiological and pathological conditions. He is currently involved in the development of a new experimental model of mouse global cerebral ischemia. He is also a member of Polish Laboratory Animal Science Association. |
| 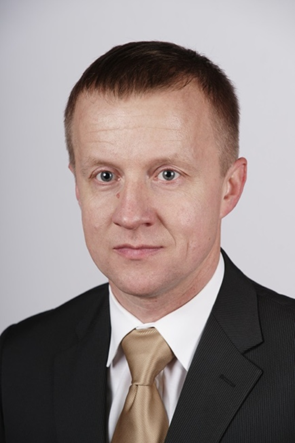 | **Artur Cichanski**, PhD Eng. graduated from the University of Science and Technology in Bydgoszcz, Poland, in 1992 in the field of Mechanical Engineering. He started to work on fatigue of materials and he defended his PhD thesis in 2000. Currently, he is an assistant professor at the University of Science and Technology in Bydgoszcz, Poland. The main field of his interest is finite element method modelling of different issues of irregular construction and material structures. |
| 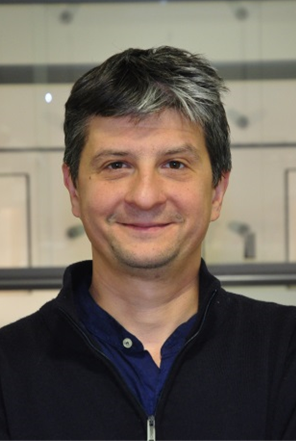 | **Maciej Wojtkowski** is active in the field of biomedical imaging. His research interest includes optical coherence tomography and low coherence interferometry applied to biomedical imaging. Dr. Wojtkowski had significant impact on development of the Fourier domain OCT (FdOCT) technique. He is an author of more than 160 publications including 90 full papers in peer reviewed journals. Currently Prof. Wojtkowski is a head of the Department of Physical Chemistry of Biological Systems at Institute of Physical Chemistry of the Polish Academy of Sciences where he also leads his own research team (Physical Optics and Biophotonics Group). |
| 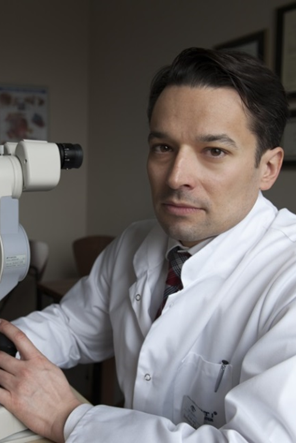 | **Bartlomiej Kaluzny**, MD, graduated from the Faculty of Medicine at the Medical Academy in Bydgoszcz, Poland in 1999. He received a PhD degree in 2005, and he finished specializing in ophthalmology in 2006. Since 2005 he has been a member of research team which constructed and performed clinical trials of the first prototype of spectral-domain OCT (SOCT). He focused on applications of SOCT in imaging of the anterior segment of the eye. His professional interests evolved from OCT to ophthalmic surgery, including cataract and refractive surgery as well as corneal transplantation. He is an author or co-author of more than 80 scientific papers and a reviewer for the journals like Ophthalmology, Cornea and Optometry and Vision Science. As a head of the Department of Ophthalmology and Optometry at Collegium Medicum NCU, Poland he is a teacher of medicine and optometry students. Part of his career was also devoted to educate and train young ophthalmologists to perform cataract and refractive surgery. |
| 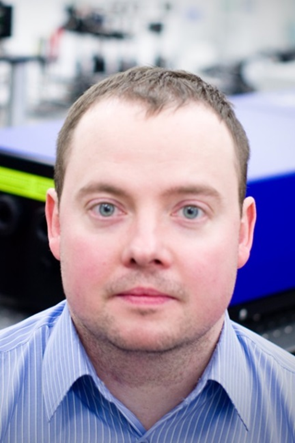 | **Ireneusz Grulkowski** received his MSc (Biomedical Physics – 2003, Biotechnology – 2005) and PhD (Experimental Physics – 2007) from the University of Gdansk, Poland. In 2010-2012, Dr. Grulkowski was a postdoctoral fellow (visiting scientist) at the Prof. J. G. Fujimoto lab at the Massachusetts Institute of Technology (Cambridge, MA, USA). He is currently an Assistant Professor at the Nicolaus Copernicus University in Torun, Poland, and the leader of the Bio-Optics and Optical Engineering Lab. The scientific interests of Dr. Grulkowski’s team include application of novel optical imaging modalities in ophthalmic diagnostics and high-resolution microscopy. Dr. Grulkowski published more than 60 articles in peer-reviewed journals and is the author of 4 chapters in books. He is a life-time member of SPIE, a member of the Optical Society of America and the Association for Research in Vision and Ophthalmology. |
