# Supplementary figures and images for: Assessment of the influence of viscoelasticity of cornea in animal ex vivo model using air‐puff optical coherence tomography and corneal hysteresis
Source: J Biophotonics. 2018 Oct 14;12(2):e201800154. doi: 10.1002/jbio.201800154 (PMC7065616; doi:10.1002/jbio.201800154)

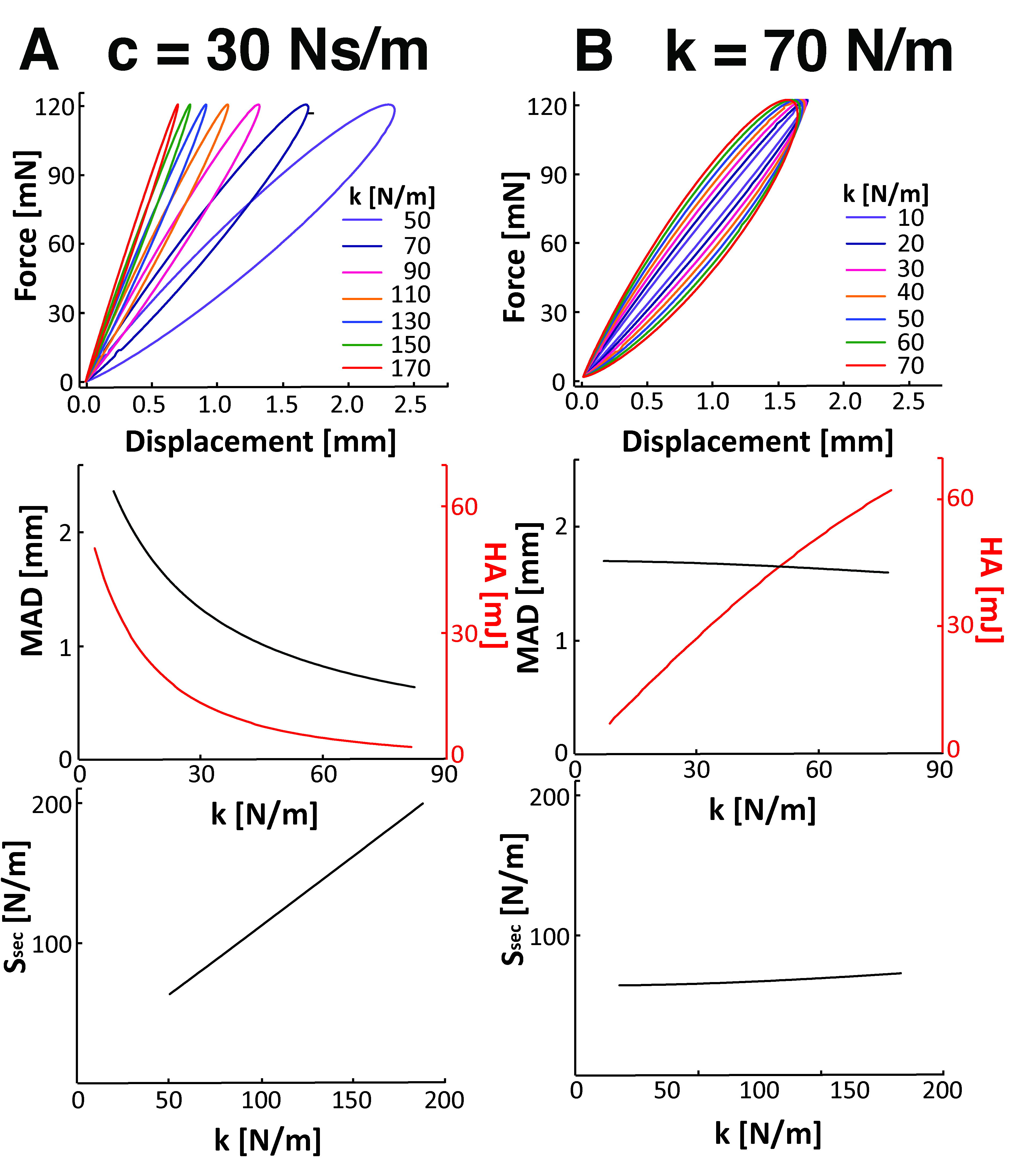

Supplement: Supplementary file 2 — FIGURE S1 Numerical results of corneal movement using Kelvin‐Voigt model of corneal viscoelasticity for different coefficients of elasticity k (A), and for different damping coefficients c (B). [file JBIO-12-e201800154-s002.tif]
